# Twin Resemblance in Muscle HIF-1α Responses to Hypoxia and Exercise

**DOI:** 10.3389/fphys.2016.00676

**Published:** 2017-01-18

**Authors:** Ruud Van Thienen, Evi Masschelein, Gommaar D'Hulst, Martine Thomis, Peter Hespel

**Affiliations:** ^1^Exercise Physiology Research Group, Department of Kinesiology, KU LeuvenLeuven, Belgium; ^2^Physical Activity, Sports and Health Research Group, Department of Kinesiology, KU LeuvenLeuven, Belgium

**Keywords:** monozygotic twin design, HIF-1, exercise, hypoxia, muscle biopsies

## Abstract

Hypoxia-inducible factor-1 (HIF-1) is a master regulator of myocellular adaptation to exercise and hypoxia. However, the role of genetic factors in regulation of HIF-1 responses to exercise and hypoxia is unknown. We hypothesized that hypoxia at rest and during exercise stimulates the HIF-1 pathway and its downstream targets in energy metabolism regulation in a genotype-dependent manner. Eleven monozygotic twin (MZ) pairs performed an experimental trial in both normoxia and hypoxia (FiO_2_ 10.7%). Biopsies were taken from m. vastus lateralis before and after a 20-min submaximal cycling bout @~30% of sea-level VO_2_max. Key-markers of the HIF-1 pathway and glycolytic and oxidative metabolism were analyzed using real-time PCR and Western Blot. Hypoxia increased HIF-1α protein expression by ~120% at rest vs. +150% during exercise (*p* < 0.05). Furthermore, hypoxia but not exercise increased muscle mRNA content of HIF-1α (+50%), PHD2 (+45%), pVHL (+45%; *p* < 0.05), PDK4 (+1200%), as well as PFK-M (+20%) and PPAR-γ1 (+60%; *p* < 0.05). Neither hypoxia nor exercise altered PHD1, LDH-A, PDH-A1, COX-4, and CS mRNA expressions. The hypoxic, but not normoxic exercise-induced increment of muscle HIF-1α mRNA content was about 10-fold more similar within MZ twins than between the twins (*p* < 0.05). Furthermore, in resting muscle the hypoxia-induced increments of muscle HIF-1α protein content, and HIF-1α and PDK4 mRNA content were about 3–4-fold more homogeneous within than between the twins pairs (*p* < 0.05). The present observations in monozygotic twins for the first time clearly indicate that the HIF-1α protein as well as mRNA responses to submaximal exercise in acute hypoxia are at least partly regulated by genetic factors.

## Introduction

Whenever the human body is exposed to oxygen deficiency, numerous physiological responses are initiated. Adaptations at both the cardiovascular, respiratory, neurological and skeletal muscle level (Petousi and Robbins, 2014) aim to maintain adequate oxygen uptake and delivery so as to preserve cellular energy homeostasis and tissue integrity. At the level of skeletal muscles, differential mechanisms are involved in the response to either acute or chronic hypoxia. For instance, in acute hypoxic stress fuel selection is shifted from fatty acids to carbohydrates, which increases the ATP yield per molecule of oxygen consumed. Such mechanism not only facilitates energy homeostasis (Hoppeler and Vogt, 2001; Hoppeler et al., 2008; Murray, 2009) but also protects against excessive mitochondrial production of reactive oxygen species (Zhang et al., 2008; Murray, 2009; Edwards et al., 2010). Furthermore, acute hypoxia also regulates gene expression of several rate-limiting enzymes in the primary energy pathways, i.e., glycolysis, the Krebs cycle, as well as oxidative phosphorylation (Zoll et al., 2006; Murray and Horscroft, 2016). These alterations eventually result in a downregulation of oxidative energy production, vs. upregulation of anaerobic ATP production via glycolysis, aiming to assure adequate rates of sustained ATP-production whenever abundant oxygen supply is lacking, most prominently during exercise (Murray, 2009). The effects of chronic hypoxia on skeletal muscle on the other hand serve to facilitate oxygen diffusion in muscle tissue by stimulation of neovascularization vs. decrease of muscle fiber cross-sectional area, which reduces oxygen diffusion distance (Hoppeler and Vogt, 2001; Deldicque and Francaux, 2013). Furthermore, loss of mitochondrial density and mitochondrial uncoupling decreased ROS production, which might otherwise be exaggerated especially during hypoxic exercise (Murray and Horscroft, 2016).

It is the prevailing opinion that hypoxia-inducible factor 1 (HIF-1) plays a pivotal role in myocellular adaptations to hypoxia (Vogt et al., 2001). HIF-1 is a heterodimeric transcription factor, built of a HIF-1α and a HIF-1β subunit, and serves as an intracellular oxygen-sensor to trigger cellular responses needed to cope with any drop of intramyocellular oxygen tension (Ameln et al., 2005; Mason and Johnson, 2007). In normoxic conditions, in contrast to hypoxia, the HIF-1α subunit is not hydroxylated and is immediately degraded. Conversely, in hypoxia, HIF-1α accumulates in the cytosol and migrates to the nucleus to dimerize with the HIF-1β subunit. The heterodimer so formed can bind to the regulatory domain of different target genes which in turn initiate the concerted cellular response to the hypoxic stress. The importance of HIF-1 in regulation of metabolic genes, including all glycolytic enzymes, *pyruvate dehydrogenase kinase 1* (PDK1) and subunit 4-2 of *cytochrome c oxidase* (COX), but also regulation of angiogenesis is well established (Ameln et al., 2005; Papandreou et al., 2006; Mason and Johnson, 2007; Murray, 2009). Activation of HIF-1 in hypoxic conditions also leads to enhanced mitochondrial autophagy and a decrease in mitochondrial biogenesis and respiration (Zhang et al., 2008). However, data on the inter-individual variability of HIF-1 responses to hypoxia and the role of heritability in HIF-1 regulation in muscle are lacking. Nonetheless, we previously found genetic variants to be important in explaining some specific muscular responses to hypoxia (regulation of maximal oxygen uptake and protein metabolism) (Masschelein et al., 2014, 2015a). Furthermore, epidemiological studies in high-altitude natives to explore the incidence of gene polymorphisms that may be beneficial for survival at high altitude, have provided evidence to indicate that the HIF transcriptional system is associated with some specific loci encoding the erythropoietin and hemoglobin proteins (Simonson et al., 2010; Yi et al., 2010; Petousi and Robbins, 2014). In contrast, the role of genetic factors in modulating the response of HIF-1 in *lowlanders* ascending to altitude is unknown. In fact, qualitative studies on the contribution of genetic factors in exercise performance in hypoxia are scarce (Hennis et al., 2015). To date published literature only supports a potential role of the *angiotensin-converting enzyme insertion* (ACE-I) and *endothelial PAS domain-containing protein 1* (EPAS1) alleles in exercise performance in hypoxia (Montgomery et al., 1998; Masschelein et al., 2015a).

Against this background, the hypothesis driving the current study was that the variability in response of myocellular HIF-1 and its downstream targets implicated in regulation of glycolysis as well as oxidative metabolism, is at least partly explained by genetic factors. To test this hypothesis, we conducted a well-controlled cross-over study (Masschelein et al., 2014, 2015b) in which monozygotic (MZ) twins were exposed to normoxia vs. hypoxia equivalent to ~5300 m altitude, both at rest and during submaximal exercise. The data presented in this paper for the first time demonstrate that both HIF-1 mRNA and protein expression are upregulated by acute normobaric hypoxia and/or exercise in a genotype-dependent manner.

## Methods

### Subjects

The data presented in this paper are original and are part of a larger study in which 13 monozygotic twin brothers (*n* = 26) were enrolled (Masschelein et al., 2014, 2015b). Inclusion criteria on admission were: non-smoking, no history of cardiovascular or respiratory disease, similar physical activity levels within twins, and no residence at altitude >1500 m during 6 months before the study. Mono-zygosity of the twin pairs was confirmed via 8 polymorphic markers (chromosome (chr) 13, GATA30H01 and GATA85D03; chr 18, GATA2E06, GATA64H04, and GATA88A12; and chr 21, GATA163G03, GATA24H09, and GATA71H10). Only 11 of the 13 twin pairs agreed to have muscle biopsies taken during the experiments and were eventually included in the current analyses (*n* = 22; age, 24.4 ± 0.8 years; body weight, 75.9 ± 1.7 kg; VO_2_max 55.3 ± 2.1 ml O_2_ kg^−1^ min^−1^). The study protocol was approved by the local ethics committee and was performed in accordance with the Declaration of Helsinki. Subjects gave written consent after being informed in detail of all experimental procedures and passed an a priori medical examination. The twins were instructed to maintain their habitual diet and physical activity levels during the study and to omit exercise for 24 h before each experimental day.

### Study design

**Figure 1:**
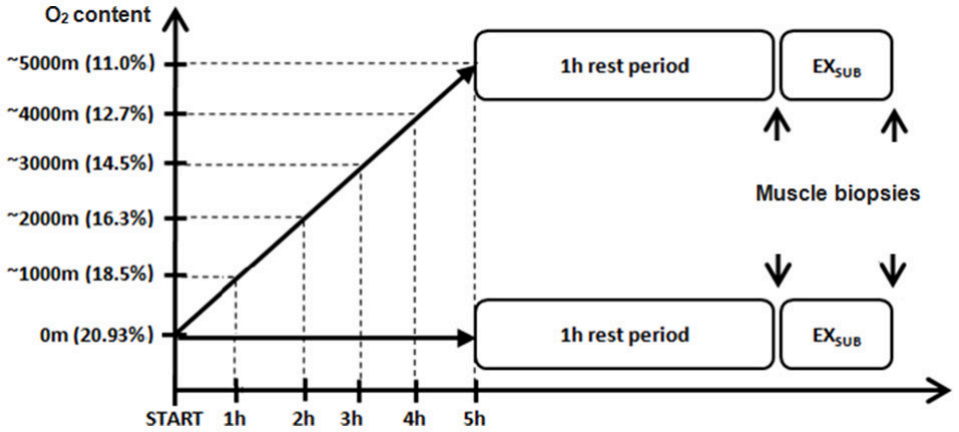
**Schematic overview of the experimental study design**. Subjects rested for 5 h in either 0.209 FIO_2_ (NOR), or while FIO_2_ was gradually decreased from 0.209 to 0.107 (HYP). Sixty min after the target FIO_2_ was established a needle biopsy sample was taken from the left m. vastus lateralis (pre-ex biopsy, see subsequent figures). Subjects then performed a 20-min submaximal constant-load (1.2 W/kg) exercise (EX_SUB_) bout on a cycle ergometer after which a second biopsy sample was obtained (post-ex biopsy, see subsequent figures). See Methods for further details.

Details of the study design have been described previously as this study is part of a greater research project (Masschelein et al., 2014, 2015b). Subjects participated in two experimental days in a normobaric hypoxic facility at 20°C and 50% relative humidity (Sporting Edge, Sherfield on Loddon, UK) with a 2-wk washout period in between. The first experimental trial was in normoxia (NOR; FIO_2_ = 0.209) and the second one in hypoxia (HYP; FIO_2_ = 0.107) (see Figure 1). The NOR experiment was always done first to exclude possible “memory” effects due to an earlier hypoxic exposure. Breakfast (700 kcal from 84% carbohydrates, 9% fat, and 7% protein) and all later meals and snacks (1880 kcal from 76% carbohydrates, 12% fat, and 12% protein) and drinks (500 ml of water per 2 h) during both the experimental days were standardized. On each day, twins reported to the 50 m^2^ hypoxic facility at 8:00 am and were put in separated compartments within the chamber in order to keep them both ignorant about the experimental events and outcomes occurring in their twin brother. They first rested for 5 h in a comfortable chair in either 0.209 FIO_2_ (NOR), or while FIO_2_ was gradually decreased from 0.209 to 0.107 (HYP). Thereafter they stayed for an additional 3 h at the target FIO_2_ of either 0.209 (NOR) or 0.107 (HYP). Sixty min after the target FIO_2_ was established a needle biopsy sample was taken from the left m. vastus lateralis under local anesthesia (1 ml of lidocaine 2 % without adrenaline) through a 5-mm incision in the skin and with the needle tip pointing proximally. Subjects then performed a 20-min submaximal constant-load (1.2 W/kg) exercise (EX) bout on a cycle ergometer (Cyclus II; Avantronic, Leipzig, Germany), corresponding to 50.7 ± 2.3% of VO_2_max in normoxia vs. 81.4 ± 3.2% of VO_2_max in hypoxia. These exercise modalities (load and duration) were selected after preliminary experiments had shown that 1.2 W/kg for 20 min in similar subjects corresponded to near-maximal exercise tolerance at 0.107 FIO_2_. Such workload (~100 W) corresponds to a normal ascent rate of 300 m per hour in mountaineers (Burtscher, 2004). We also wanted to compare responses to an absolute workload rather than a relative workload. Immediately after exercise (post-ex), another muscle biopsy sample was taken through the same incision as the pre-ex biopsy sample but with the needle pointing distally in the muscle. All muscle samples were quickly frozen in liquid nitrogen and stored at −80°C until biochemical assays were performed.

### Western blot

Details of the immunoblotting procedures were described previously (Deldicque et al., 2010). Briefly, frozen muscle tissue (~20 mg) was homogenized 3 times for 5 s each with a Polytron mixer (Polytron Technologies, Taoyuan City, Taiwan) in ice-cold buffer [1:10, w/v; 50 mM Tris-HCl, pH 7.0; 270 mM sucrose; 5 mM EGTA; 1 mM EDTA; 1 mM sodium orthovanadate; 50 mM glycerophosphate; 5 mM sodium pyrophosphate; 50 mM sodium fluoride; 1 mM dithiothreitol; 0.1% Triton X-100; and a complete protease inhibitor tablet (Roche Applied Science, Vilvoorde, Belgium)]. Homogenates were then centrifuged at 10,000 g for 10 min at 4°C. The supernatant was collected and immediately stored at −80°C. The protein concentration was measured using a DC protein assay kit (Bio-Rad Laboratories, Nazareth, Belgium). Proteins (30–80 g) were separated by SDS-PAGE (8–12% gels) and transferred to polyvinylidene difluoride membranes. Subsequently, membranes were blocked with 5% nonfat milk for 1 h and then incubated overnight (4°C) with the following antibodies (1:1000): total eukaryotic elongation factor 2 (eEF2) (Cell Signaling, Leiden, The Netherlands) and hypoxia-inducible factor-1α (HIF-1α). Horseradish peroxidase-conjugated anti-rabbit (1:5000) and anti-guinea pig (1:5000) secondary antibodies (Sigma-Aldrich, Bornem, Belgium) were used for chemiluminescent detection of proteins. Membranes were scanned and quantified with GeneSnap and Gene Tools software (Syngene, Cambridge, UK), respectively. The results are presented as the ratio protein of interest/eEF2. All values from the respective condition were reported to the mean value of the first sample (pre-ex) in NOR. One subject was excluded from analysis due to lack of sample.

### RNA extraction and reverse transcription

The method used for reverse transcription is described in detail elsewhere (Vincent et al., 2010; Jamart et al., 2011). In brief, total RNA was extracted using TRIzol (Invitrogen, Vilvoorde, Belgium) from 20 to 25 mg of frozen muscle tissue. Total RNA was extracted only in 19 subjects because of the lack of material in 3 individuals. RNA quality and quantity were assessed by spectrophotometry with a NanoDrop (Thermo Scientific, Erembodegem, Belgium). Then 1 g of RNA was reverse-transcribed using a High-Capacity cDNA Reverse Transcription kit (Applied Biosystems, Gent, Belgium) according to the manufacturer's instructions.

### Real-time quantitative PCR analysis

A SYBR Green-based master mix (Applied Biosystems) was used for real-time PCR analyses using the ABI PRISM 7300 system (Applied Biosystems). Real-time PCR primers were designed for the genes of interest. Thermal cycling conditions consisted of 40 3-step cycles including denaturation for 30 s at 95°C, annealing for 30 s at 58°C, and extension for 30 s at 72°C. All reactions were performed in triplicate. To compensate for variations in input RNA amounts and efficiency of reverse transcription, glyceraldehyde-3-phosphate dehydrogenase (GAPDH) and ribosomal protein L4 (RPL4) mRNA were quantified, and results were normalized to these values. These genes were chosen out of 3 normalization genes using the GeNorm applet according to the guidelines and theoretical framework described elsewhere as the Vandesompele-method (Vandesompele et al., 2002). All values from the respective condition were reported to the mean value of the first sample (pre-ex) in NOR.

### Statistical analysis

A 2-way repeated-measures analysis of variance (ANOVA) was used to assess the statistical significance of differences between mean values over time and between conditions (Systat Software, San Jose, CA, USA). When appropriate, a Bonferroni *t*-test was used as a *post hoc* test. In addition, the correlated observations at the level of the twins, together with the individual repeated measures of the oxygen level conditions (NOR vs. HYP) and exercise response (time), were also analyzed using a multilevel mixed-model approach (random effects for time level nested in condition level, nested in twin level; SAS 9.3; SAS Institute, Cary, NC, USA). Because the multilevel mixed-model approach gave the same significant results as the ANOVA, it was chosen to present all data according to the results of the 2-way repeated-measures ANOVA analyses. When the latter analyses revealed a significant difference in protein or mRNA expression, the genetic influence was determined by 2-way repeated-measures ANOVA on one factor, with the twins nested in pairs, and by intra-class correlation coefficients (ICC's; SAS Enterprise Guide 4.3, SAS Institute). F-ratios thus obtained represent the ratio of between-pair over within-pair variability in the induced responses, whereas ICCs provide a quantitative estimate of the similarity within MZ twin pairs and an upper-limit estimate of the genetic component in these responses (genotype × hypoxia and genotype × exercise interaction) (Bouchard et al., 1990). A probability level of *P* < 0.05 was considered statistically significant. All data are presented as means ± standard of the mean (SEM).

## Results

### Hif-1α pathway (Table 1 and Figures 2–4)

Compared to NOR, HYP increased HIF-1α protein expression by ~120% at rest, and by an additional 35% post exercise (*p* < 0.05). Compared to the resting condition in NOR, exercise in HYP raised HIF-1α protein expression about 2.5-fold (*p* < 0.05). HYP, but not exercise, increased HIF-1α mRNA expression by about 30–50% (*p* < 0.05). Irrespective of rest and exercise, HYP stimulated PHD2 as well as pVHL mRNA content by ~40–50% (*p* < 0.05), whilst PHD1 mRNA content was affected neither by HYP, nor by exercise. Compared to NOR, HYP slightly elevated VEGF-2A mRNA content post (*p* < 0.05) but not pre exercise.

**Table 1:** **MZ twin resemblance for muscle protein and mRNA responses to hypoxia and/or exercise**.

	**Effect of hypoxia**				**Effect of exercise**			
	**Pre-ex NOR vs. pre-ex HYP**		**Post-ex NOR vs. post-ex HYP**		**Pre-ex NOR vs. post-ex NOR**		**Pre-ex HYP vs. post-ex HYP**	
	**ICC**	***F***	**ICC**	***F***	**ICC**	***F***	**ICC**	***F***
**HIF-1**α **PATHWAY**								
HIF-1α (protein)	0.72^*^	2.53^*^	0.45	0.82	0.23	0.29	0.58	1.37
HIF-1α (mRNA)	0.79^*^	3.77^*^	0.91^*^	9.80^*^	0.44	0.78	0.89^*^	8.29^*^
PHD1	0.46	0.86	0.51	1.03	0.85^*^	5.50^*^	0.51	1.04
PHD2	0.63	1.68	0.58	1.38	0.70	2.30	0.48	0.94
pVHL	0.52	1.07	0.74^#^	2.84^#^	0.52	1.10	0.83^*^	4.81^*^
VEGF-2a	0.79^*^	3.82^*^	0.67	2.04	0.49	0.94	0.76^#^	3.10^#^
**GLYCOLYTIC METABOLISM**								
PFK-M	0.60	1.50	0.56	1.29	0.67	2.04	0.61	1.58
PDH-A1	0.62	1.63	0.49	0.95	0.77^#^	3.30^#^	0.62	1.65
PDK4	0.77^#^	3.43^#^	0.78^*^	3.56^*^	0.68	2.08	0.11	0.13
LDH-A	0.72^#^	2.62^#^	0.45	0.82	0.46	0.85	0.84^*^	5.17^*^
**OXIDATIVE METABOLISM**								
CS	0.48	0.93	0.58	1.39	0.69	2.18	0.70	2.29
COX-4	0.44	0.80	0.59	1.45	0.55	1.20	0.59	1.42
PGC-1α	0.33	0.50	0.74^#^	2.89^#^	0.75^#^	2.98^#^	0.82^*^	4.46^*^
PPAR- γ1	0.77^#^	3.37^#^	0.42	0.73	0.46	0.85	0.78^#^	3.65^#^
TFAM	0.64	1.77	0.27	0.37	0.59	1.42	0.53	1.13

*ICC's and corresponding F-ratios (between/within pair's variance; n = 11) represent similarity within twin pairs for the hypoxia response either pre or post exercise (ex), or similarity within twin pairs for the exercise response during either NOR or HYP. Individual twin-pair responses for the underscored results are presented in Figure 4*.

* *P < 0.05*;

# *P < 0.10*.

**Figure 2:**
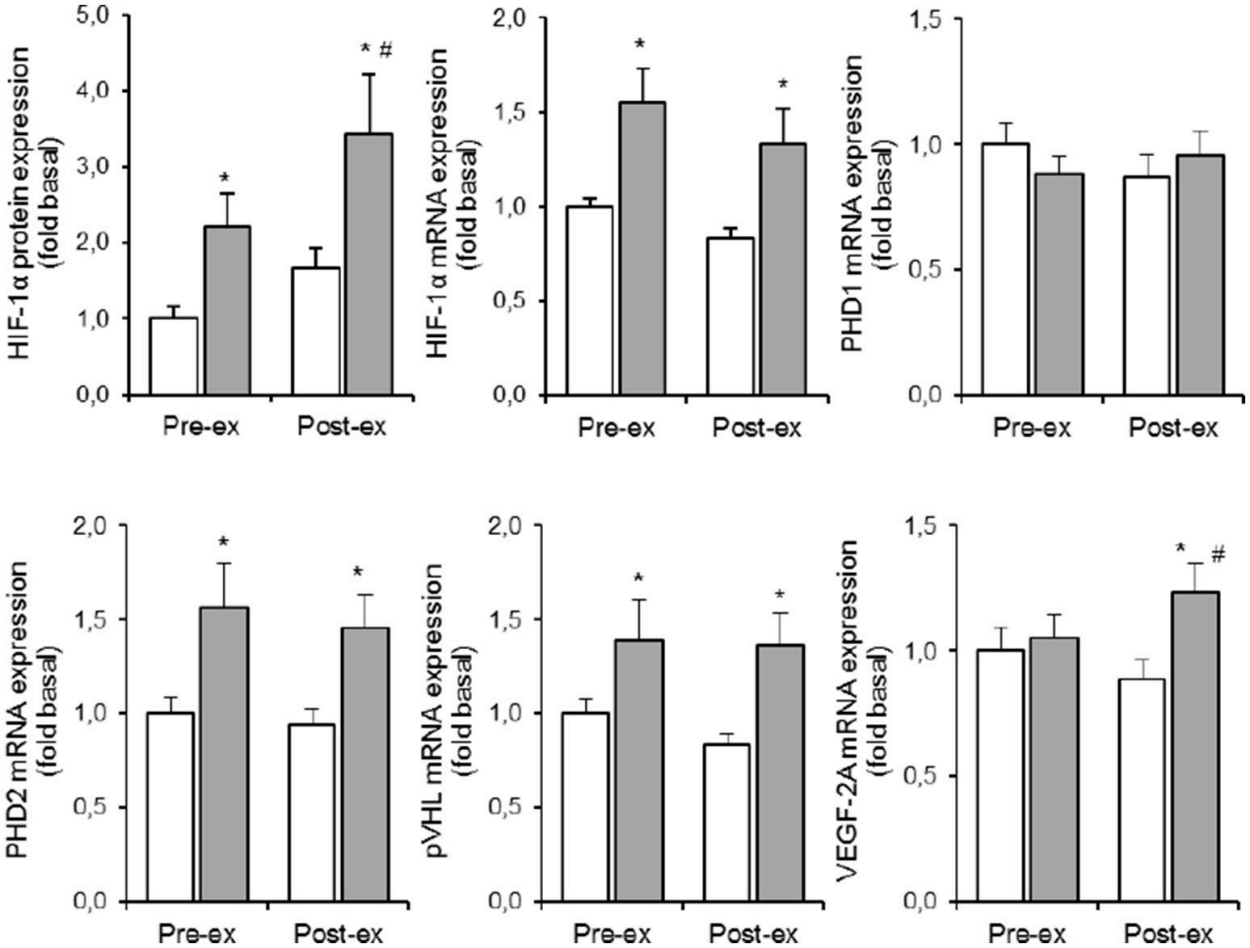
**Effect of hypoxia and exercise on the HIF-1α pathway**. Data are means ± SEM of 11 twin pairs (*n* = 22) before (pre-ex) and immediately after (post-ex) a 20-min submaximal exercise bout in either normoxia (empty bars) or hypoxia equivalent to ~5300 m altitude (full bars). HIF-1α, hypoxia-inducible factor 1α; PHD1-2, prolyl hydroxylase domains 1-2; pVHL, Von Lippel-Hindau protein; VEGF-2A, vascular endothelial growth factor-2A. See Methods for further details. ^*^P < 0.05 vs. NOR ^#^*P* < 0.05 vs. pre-ex.

Some of the above average responses were not randomly distributed between subjects. Conversely, some responses were significantly more homogeneous within the twins than between the twins (Table 1). Most strikingly, stimulation of HIF-1α mRNA content by the combination of exercise and HYP yielded high similarity between twin brothers (see **Figure 4**). The effect of HYP to stimulate the exercise-induced increment in HIF-1α mRNA content was about 10-fold more homogeneous within twin pairs than between twin pairs (*p* < 0.05), with an ICC as high as 0.91 (*p* < 0.05). By analogy, the effect of exercise to stimulate HIF-1α in HYP, showed about 8-fold higher similarity within the twin pairs (ICC: 0.89, *p* < 0.05) than between the twins. Significant twin resemblance was also found for the effect of HYP to raise HIF-1α protein (ICC = 0.72, *p* = 0.05, Figure 2) and mRNA (ICC = 0.79, *p* < 0.05, Figure 2) content in resting muscle. However, the effect of exercise, alone or in combination with HYP, to stimulate HIF-1α protein content did not yield significant twin resemblance. Furthermore, the exercise-induced increment of pVHL mRNA content in HYP, but not in NOR, was ~5-fold more similar within twins than between twins (ICC = 0.83, *p* < 0.05).

### Glycolytic metabolism (Table 1 and Figure 5)

**Figure 3:**
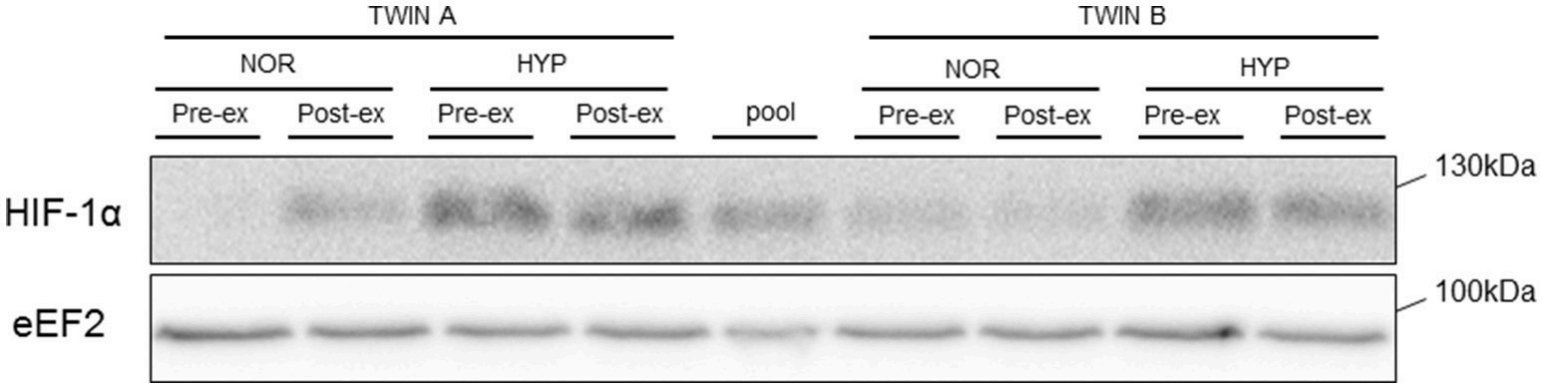
**Representative blot of HIF-1α protein**. Representative blot of HIF1-α in one monozygotic twin pair before (pre-ex) and immediately after (post-ex) exercise in either normoxia (NOR) or hypoxia (HYP). eEF2 is shown as loading control.

Compared to NOR, HYP increased PDK4 mRNA expression ~12-fold both pre and post exercise (*p* < 0.05; Figure 3). In the resting condition in HYP, mRNA levels of PFK-M were ~20% higher compared to NOR with no additional effect of exercise (*p* < 0.05; Figure 3). Neither LDH-A nor PDH-A1 mRNA expression was affected by either exercise or hypoxia (**Figure 5**).

The HYP-induced increased PDK4 gene expression was ~3.5-fold more similar within twin brothers than between the different twin pairs (*p* < 0.05). Furthermore, exercise in HYP on average did not significantly increase LDH-A mRNA content indeed, yet responses were ~5-fold more similar within twins than between twins. Overall variability in PFK-M mRNA expression on the other hand, was comparable between subjects with no significant twin resemblance.

### Oxidative metabolism (Table 1 and Figure 6)

**Figure 4:**
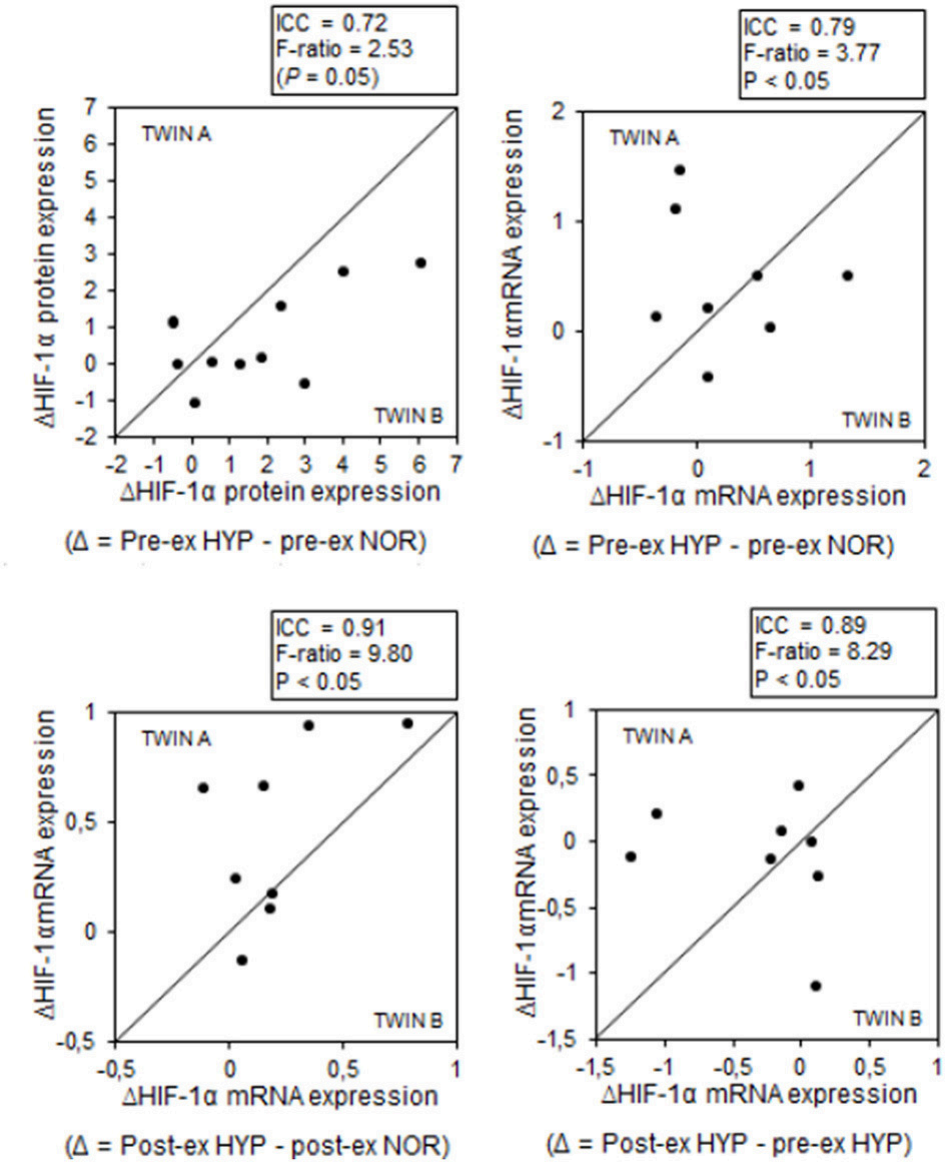
**MZ twin resemblance for the effects of hypoxia and exercise on the HIF-1α pathway**. Each data point represents the values for one pair of twins (twin A vs. twin B) before (pre-ex) and after (post-ex) a 20-min submaximal exercise bout. Data are hypoxia-induced changes (Δ) and exercise-induced changes for either HIF-1α protein or mRNA expression. Intra-class correlations and corresponding F-ratios (between/within pair's variance) are given in the figure. See Methods for further details.

HYP increased PPAR-γ1 mRNA expression to the same degree (+60%, *p* < 0.05, **Figure 6**) both at rest and during exercise. Pre exercise PGC-1α mRNA levels were identical in NOR and HYP. However, exercise decreased PGC-1α mRNA expression by ~15% in NOR (*p* < 0.05), vs. increased PGC-1α mRNA content by ~15% in HYP (*p* < 0.05, Figure 4). TFAM responses to exercise in hypoxia were comparable, which resulted in higher TFAM mRNA content post exercise in HYP compared with NOR (*p* < 0.05, Figure 4). Neither exercise nor HYP affected either COX-4 or CS (**Figure 6**) mRNA contents.

The response of PGC-1α mRNA expression to exercise was about 3 to 5-fold more similar within twin pairs than between twin pairs, particularly during exercise in HYP (*p* < 0.05). Conversely, variability of either exercise-induced or hypoxia-induced changes of citrate synthase, COX-4, PPAR-γ, and TFAM mRNA expressions were not significantly different between twins than within twin pairs.

## Discussion

In this study we explored the contribution of genetic factors in the myocellular responses of HIF-1α and its downstream targets to acute hypoxia. We exposed monozygotic twin brothers to high altitude (~5300 m) simulated by means of normobaric hypoxia (FIO_2_ 0.107) and compared the responses with normoxia (FIO_2_ 20.9%), both at rest and during submaximal exercise. We have previously reported that the current protocol on average decreased VO_2_max by ~40% (range: 25–55%), yet with very high twin resemblance (Masschelein et al., 2015b). Furthermore, we also found hypoxia to modulate protein metabolism at rest and after moderate exercise by increasing markers of protein breakdown, more specifically markers of the autophagy-lysosomal system, including a significant genetic contribution for both hypoxia-induced regulation of REDD1 and microtubule-associated protein 1 light chain 3 (LC3) lipidation (Masschelein et al., 2014). Here we postulated that if heritability is an important determinant of myocellular adaptation to hypoxia, then this must translate into genotype-dependent regulation of HIF-1α during an acute hypoxic challenge. In support of such contention, our current data for the first time clearly demonstrate that acute hypoxia, alone or in conjunction with exercise, substantially increased both muscle HIF-1α mRNA and protein content. Moreover, the HIF-1α increments yielded much higher within-twin than between-twin resemblance, indicating significant genotype impact.

Hypoxia-inducible factor-1α (HIF-1α) plays an important role in the acute responses as well as in the long-term cellular adaptations to oxygen deficiency (Stroka et al., 2001). Upon decreasing intracellular PO_2_, HIF-1α translocates to the nucleus to dimerize with the constitutively active HIF-1β. The HIF1 complex so formed eventually acts as a transcription factor for a larger number of genes implicated in regulation of muscle energy metabolism and morphology (Zhang et al., 2008; Luo et al., 2011). In tumor cells, ATP production was impaired and HIF-1α-dependent genes were upregulated whenever intracellular PO_2_ dropped below ~10 mmHg (Richardson et al., 2006; Flueck, 2009). Conversely, muscle cells appear to be more resistant to oxygen deficit because the rate of ATP production is well maintained even at <10 mmHg intracellular PO_2_ values inherent to high-intensity muscle contractions (Richardson et al., 1995, 2006; Favier et al., 2015). Conversely, at the very low rates of ATP turnover existing in resting muscle a net arterial PO_2_ drop by at least 30 mmHg is needed to significantly decrease intramyocellular PO_2_ (Johnson et al., 2005; Richardson et al., 2006). Accordingly, even at an inspired PO_2_ as low as ~75 mmHg corresponding to ~5500 m altitude, muscle PO_2_ is maintained at ~20–25 mmHg, which is still manifold higher than during maximal exercise in normoxia (<5 mmHg) (Richardson et al., 1995, 2006). Based on the above observations it has been postulated that passive exposure to a low-oxygen environment cannot elicit HIF stabilization (D'Hulst et al., 2013; Favier et al., 2015). Our current observations clearly contradict such conclusion. We did not measure intracellular PO_2_, indeed, yet in line with earlier observations (Richardson et al., 2006; Rissanen et al., 2012; D'Hulst et al., 2013) resting arterial oxygen saturation on average decreased from ~98% in normoxia to ~78% in 0.107 FIO_2_ (Masschelein et al., 2015b), which should yield intracellular PO_2_ values in the range of ~20–25 mmHg (Richardson et al., 2006). Nonetheless, HIF-1α protein expression on average increased ~ 2-fold and further increased during exercise (20 min @1.2 W/kg; see Figure 2). This submaximal exercise alone did not stimulate HIF-1α protein expression. To the best of our knowledge, this is the first experiment documenting HIF-1α stabilization due to ambient hypoxia in healthy humans at rest. It is difficult to explain the discrepancy between our current and previous observations (D'Hulst et al., 2013). However, besides the rate and magnitude of effected intramyocellular PO_2_ decrease, also training status (Lindholm et al., 2014) and history of hypoxic exposure conceivably play a role in regulation of HIF-1α stabilization.

We have previously shown by using near-infrared spectroscopy that normobaric hypoxia equivalent to 5300 m decreased muscle tissue oxygenation by no more than ~8% compared to normoxic conditions (Masschelein et al., 2014; Van Thienen and Hespel, 2016). Addition of a submaximal cycling exercise bout similar to the present protocol, decreased oxygenation status by an additional ~12 vs. 5% in normoxia (Masschelein et al., 2014; Van Thienen and Hespel, 2016). These data indicate that the effects of hypoxia and exercise on muscular oxygenation status are additive. Still, tissue oxygenation status measured by near-infrared spectroscopy only partially reflects intramyocellular oxygen tension, which is critical to regulation of HIF-1α. It has been shown that stabilization and expression of HIF-1α is tightly regulated by decreases in intramyocellular PO_2_ within the physiological range. Acute exercise reduces myocellular PO_2_ to 1/40th of that of inhaled air (PIO_2_) while intramyocellular PO_2_ values at rest remain at ~1/5th of the PIO_2_ (Richardson et al., 1995). In the conditions of the current study, PIO_2_ in hypoxia was ~75 mmHg which conceivably made intramyocellular PO_2_ to drop below 2 mmHg, which is ample to elicit HIF-1α stabilization (Richardson et al., 1995, 2006; Fong and Takeda, 2008). Indeed, due to the specific value of the Michaelis constant of ~240 μM, the hydroxylase activity of PHD2 is dependent and sensitive to oxygen partial pressures in the range of 0.1–30 mmHg typically occurring in muscle cells at rest and during exercise (Richardson et al., 1995). As the inhibition of PHD2, and thus stabilization of HIF-1α is proportional to the degree of intracellular PO_2_ drop (Fong and Takeda, 2008), hypoxia and exercise logically act as additive agents to stimulate HIF-1α protein stabilization and expression via inhibition of PHD2 activity. Furthermore, literature data indicate that HIF-1 is not only regulated by decrease in myocellular PO_2_ but also through some humoral factors such as α-ketoglutarate. Plasma α-ketoglutarate concentration increases due to elevated Krebs cycle activity during exercise (Leibowitz et al., 2012). Available evidence indicates that α-ketoglutarate can stabilize HIF-1, probably via inhibition of the PHD2 enzyme (Hou et al., 2014). This provides a mechanism by which exercise *per se*, but not hypoxia, can stimulate HIF-1 activity. Thus, exercise in hypoxia will stimulate HIF-1 activity more than exercise or hypoxia alone, due to Ameln et al. (2005) the synergistic effects of exercise and hypoxia in decreasing myocellular PO_2_ and thus activation of PHD2 and (Aragonés et al., 2009) the additional effect of exercise-induced α-ketoglutarate over hypoxia-induced HIF-1 stabilization.

The current hypoxic protocol caused a consistent stimulation of HIF-1α, indeed, yet the responses were highly variable between individuals. Individual changes ranged from −100 to +600% for HIF-1α protein content, vs. no change to +150% for mRNA expression. Interestingly, however, the HIF-1α responses exhibited high twin resemblance. F-ratios indicate that inter-pair variability was about 10-fold greater than intra-pair variability (see Figure 4), and hypoxia-induced changes of both muscle HIF-1α protein (ICC = 0.72) and mRNA (ICC = 0.91) contents yielded high MZ twin correlations. In addition, ICCs were substantially higher for the hypoxia effects compared to the exercise effects. This clearly indicates genetic modulation of muscular HIF-1α responses during acute hypoxic stress. Further support for such statement comes from the observation that the hypoxia-induced, but not the exercise-induced changes in mRNA expression of VEGF-2α, a primary downstream target of HIF-1α, were highly similar within twin brothers (ICC = 0.76). In addition, the responses of VEGF-2α were ~3-fold more variable between twin-pairs than within the pairs. In line with published literature data (Ke and Costa, 2006; Fong and Takeda, 2008), we also observed hypoxia-induced stabilization of muscle HIF-1α to be associated with increased mRNA expression of PHD2 and pVHL. However, for these regulators of HIF-1α degradation we could not demonstrate genotype-dependence, which may be at least partly explained by predominant regulation of the HIF-1 pathway at the post-transcriptional level (Aragonés et al., 2009; Formenti et al., 2010). The increase in the mRNA expression of PHD2 and pVHL may seem counter intuitive. However, it has been shown before that acute hypoxia selectively increases expression of both PHD2 mRNA and protein levels in rat glioma cells and in cell cultures (Berra et al., 2003; D'Angelo et al., 2003). This hypoxic upregulation of PHD2 probably acts as a negative feedback loop to immediately stop hypoxic and thus HIF-1 responses in cells once they are re-oxygenated again (Berra et al., 2003; D'Angelo et al., 2003).

**Figure 5:**
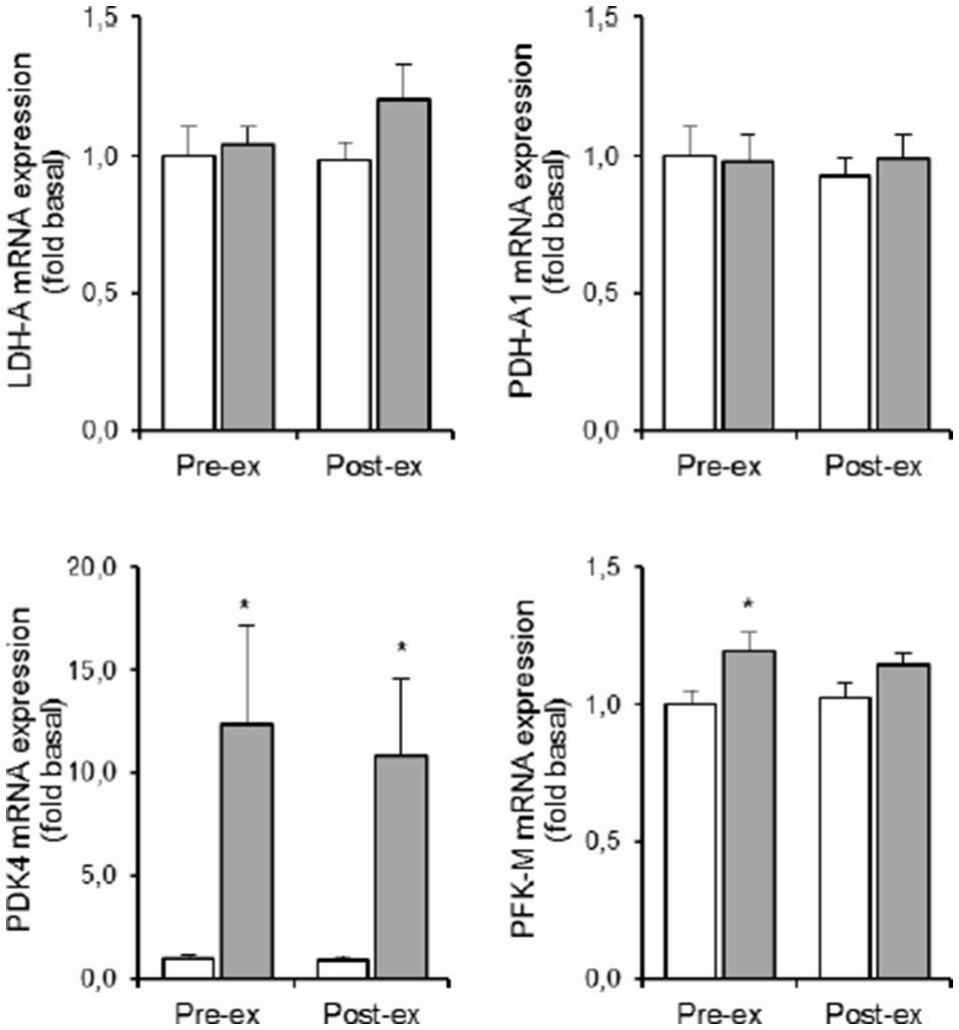
**Effect of hypoxia and exercise on glycolytic metabolism**. Data are means ± SEM of 11 twin pairs (*n* = 22) before (pre-ex) and immediately after (post-ex) a 20-min submaximal exercise bout in either normoxia (empty bars) or hypoxia equivalent to ~5300 m altitude (full bars). LDH-A, lactate dehydrogenase-A; PDH-A1, pyruvate dehydrogenase-A1; PDK4, pyruvate dehydrogenase kinase-4; PFK-M, phosphofructokinase muscle-type. See Methods for further details. ^*^P < 0.05 vs. NOR.

Long-term acclimatization to altitude/hypoxia a.o. involves metabolic reprogramming yielding higher glycolytic capacity to compensate for reduced rate of oxidative phosphorylation during episodes of explicit O_2_-deficiency, e.g., during contractions (Harris et al., 2002). The pyruvate dehydrogenase (PDH) enzyme-complex channels the output of pyruvate units from glycolysis into either acetyl-CoA formation, or into the lactate dehydrogenase reaction to produce ATP via lactate production. PDK4 inhibits muscle PDH activity by phosphorylation (Harris et al., 2002; Kim et al., 2006; Lee et al., 2012). It has been demonstrated that HIF-1α stimulates PDK4 gene transcription via regulation of the estrogen-related nuclear receptor (Lee et al., 2012). We did not measure ERR activation, yet in keeping with the above pathway of PDK4 activation we found hypoxia-induced HIF-1α stabilization to be associated with a substantial increment of muscle PDK4 gene transcription rate (~10-fold increased mRNA content; see Figure 5). Assuming that repeated hypoxic exposures eventually will upregulate PDK4 enzyme activity, energy provision via anaerobic glycolysis is likely to be facilitated due to inhibition of PDH-A1 activity. Interestingly, the hypoxia-induced change in PDK4 mRNA expression also exhibited high MZ twin resemblance (ICC = 0.77), indicating that PDK4 also is a site of genotype-dependent regulation of acute hypoxic adaptation. Additional support for upregulation of the glycolytic pathway comes from our observation that hypoxia *per se* also slightly elevated muscle PFK mRNA content.

**Figure 6:**
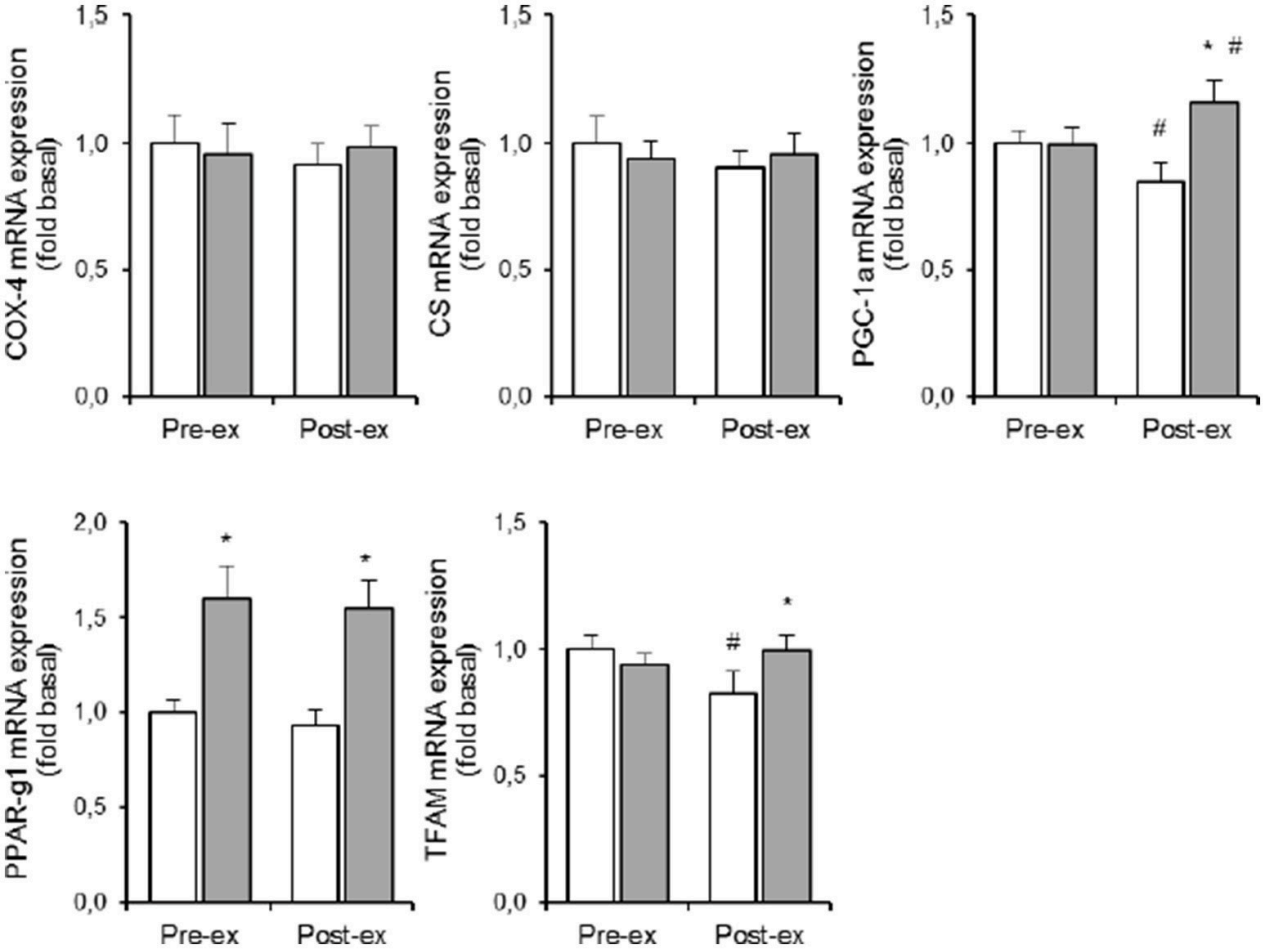
**Effect of hypoxia and exercise on oxidative metabolism**. Data are means ± SEM of 11 twin pairs (*n* = 22) before (pre-ex) and immediately after (post-ex) a 20-min submaximal exercise bout in either normoxia (empty bars) or hypoxia equivalent to ~5300 m altitude (full bars). COX-4, cytochrome c oxidase-4; CS, citrate synthase; PGC-1A, peroxisome proliferator-activated receptor gamma, coactivator 1A; PPAR-γ1, peroxisome proliferator-activated receptor gamma-1; TFAM, mitochondrial transcription factor-A. See Methods for further details. ^*^P < 0.05 vs. NOR ^#^*P* < 0.05 vs. pre-ex.

The PGC-1 family of regulated coactivators plays a pivotal role in the control of mitochondrial biogenesis and respiratory function. PGC-1-induced upregulation of mitochondrial mass increases the capacity for oxygen consumption, which in turn leads to drop of intracellular oxygen availability and stabilization of HIF-1 (O'Hagan et al., 2009). However, such chronic adaptive mechanism is irrelevant during acute hypoxia, which in the conditions of the current study did not even elevated muscle PGC-1α mRNA content. However, PGC-1 activity which is largely regulated by posttranslational modifications which in turn tune the activity of a series of downstream targets, including peroxisome proliferator-activated receptor-γ, which also modulates PDK4 protein expression (Wende et al., 2005). Acute hypoxia here raised PPAR-γ1 mRNA level by ~60% (Figure 6), and again changes were highly similar within the MZ twins (ICC = 0.78, F-ratio = 3.65; *p* < 0.05). This finding adds evidence to indicate that genetic background plays a role in upregulation of anaerobic glycolysis via stimulation of the PPAR-γ~PDK4~PDH pathway in hypoxia.

Because long-term exposure to sever hypoxia decreases mitochondrial volume density in muscle cells (Hoppeler et al., 2003), we also measure some key-enzymes and nuclear transcription factors that are implicated in mitochondrial metabolism and biogenesis. However, 5 h of acute exposure to simulated ~5300 m altitude changed neither CS nor COX-4 mRNA expression, which is in line with published literature (Edwards et al., 2010; Horscroft and Murray, 2014; Murray and Horscroft, 2016). Also muscle mRNA contents of PGC-1 and TFAM were unchanged, and in addition data variability was small. Hence our current set-up does not allow to evaluate the genetic contribution in hypoxia-induced regulation of oxidative metabolism. Moreover, the timing of the muscle biopsies may have been inadequate to detect elevated mRNA expressions for some of the aforementioned variables.

Because of obvious limitations in finding twin pairs eligible to participate in our invasive study, we chose to recruit only monozygotic twins in order to accumulate a significant number of observations within this specific group. Similar monozygotic twin study designs previously were successful to explore genotype~environment interactions in phenotype adaptation (Bouchard et al., 1986, 1990). Nonetheless, because we did not include dizygotic twins in the study design, it is not possible to discriminate here between genetic and shared environmental similarity-inducing factors (Bouchard et al., 1990). For MZ twin resemblance results from both true genetic and shared family environmental effects, at least if both members of the pair were raised/lived together. Therefore, twin resemblances reported here probably reflect the upper-limit of heritability. Nonetheless, one should also recognize that data variability inherent to the muscle biopsy procedure *per se* (Van Thienen et al., 2014) as well as rtPCR and Western Blotting assays, confound the true physiological variabilities within and between twins. Therefore, the physiological significance of some upper-limit heritability coefficients may be even higher than could appear from the numerical statistical output. Accordingly, the absence of statistically significant ICC's and/or F-ratio's does not exclude genotype-dependent regulation. For the average effect-size and concurrent data variability for some variables might have been too small, indeed, to allow pertinent correlational analyses to compare within-twin and between-twin variabilities. This study has shown genotype^*^exercise and genotype^*^hypoxia interactions to exist for the HIF-1α pathway as well for its downstream transcriptional targets. Future research should aim to identify specific gene variants using genome-wide association studies.

In conclusion, our study provides novel data to prove that genetic factors play an important role in the muscular responses to acute hypoxic stress at rest and during exercise in young healthy individuals. We for the first time clearly show that regulation of HIF-1α stabilization in acute hypoxia is genotype-dependent. This report therefore contributes to a better understanding of the variation in the integrated response to altitude/hypoxia in humans.

## Author contributions

RVT and EM: Conception and design of research, prepared figures and manuscript, edited and revised manuscript, approved final version of manuscript. GD: Analyzed results of experiments, edited and revised manuscript, approved final version of manuscript. MT and PH: Conception and design of research, edited and revised manuscript, approved final version of manuscript.

## Funding

This study was supported by grant OT/09/033 from the Katholieke Universiteit Leuven.

### Conflict of interest statement

The authors declare that the research was conducted in the absence of any commercial or financial relationships that could be construed as a potential conflict of interest.
